# Long-term outcome after middle third fractures of the femur shaft; a comparison between antegrade and retrograde nailing

**DOI:** 10.1007/s00068-025-02990-9

**Published:** 2025-10-28

**Authors:** J. Daniël Cnossen, Esther M. M. Van Lieshout, Michael H. J. Verhofstad

**Affiliations:** Trauma Research Unit Department of Surgery, Erasmus MC, University Medical Center Rotterdam, P.O. Box 2040, Rotterdam, 3000 CA the Netherlands

**Keywords:** Femoral shaft fracture, Intramedullary nailing, Rotational malalignment, Functional outcome, Treatment outcome

## Abstract

**Purpose:**

This study compared patient-reported functional outcomes, range of motion, and radiological alignment in antegrade (AN) versus retrograde nailing (RN) for midshaft femur fractures (AO/OTA type 32).

**Methods:**

A retrospective review of adult patients treated between January 1, 2001, and May 1, 2016, was conducted. Patients completed questionnaires and underwent physical exams to measure leg length, rotation, and hip/knee range of motion. Final radiographs assessed union alignment.

**Results:**

A total of 162 patients with 173 fractures were included, with a median follow-up of 8.2 years. Of the 52 patients completing full follow-up, 8% showed rotational malalignment with a flexed hip, and 6% had > 15° malalignment with an extended hip. Knee flexion-extension range was slightly better in the AN group (130° vs. 125°; *p* = 0.019). Patients treated with AN also showed better results in the Lower Extremity Functional Scale (LKS) (83 vs. 61; *p* = 0.041) and the SMFA dysfunction index (13 vs. 29; *p* = 0.026) compared to RN.

**Conclusion:**

While both nailing techniques produced similar overall outcomes, AN demonstrated fewer complications, fewer rotational issues, and better patient-reported outcomes. Based on these findings, AN is preferred over RN for treating midshaft femoral fractures when technically feasible.

## Introduction

Femoral shaft fractures are mostly caused by high energy trauma (HET) and occur frequently in the young [1, 2]. Intramedullary nailing has evolved as the golden standard for definitive fracture repair since it is a minimally invasive technique providing the ability to exercise and mobilize early [3–5].

An intramedullary nail can be inserted both antegrade or retrograde, each with assumed benefits and risks. Reported disadvantages of antegrade nailing of the femur include the risk of injury to the hip abductors or its vascular and nerve supply [6–10]. Retrograde nailing for femoral shaft fractures is attractive in certain situations, such as polytrauma, ipsilateral fractures of the lower leg, obese patients, or a distal femoral shaft fracture [9, 11]. Retrograde nailing requires an articular approach and may result in complications of the knee joint, including infection, damage to the articular cartilage, decrease in range of motion of the knee, and persistent knee pain [12–14].

The use of entry points that do not match the design of the nail or inadequate fracture reduction can cause deformities, which can lead to patient discomfort. An angular malalignment >5° and rotational malalignment of >20° are considered clinically relevant and might justify surgical correction.

Various studies on both nailing techniques in femoral shaft fractures showed divergent results in patient reported outcome and range of motion [9, 11, 12, 15, 16]. However, fractures in the periphery of the shaft might benefit from angular stable fixation in the short fragment and thus favor one of both techniques. Moreover, such a peripheral shaft fracture can be accompanied by injuries to the adjacent hip and knee joint and attribution of final outcome to the injury or the surgical technique can be difficult. The middle-third fracture of the femur shaft is an injury that can technically be addressed both retrograde and antegrade with an assumed similar biomechanical result.

**Fig. 1:**
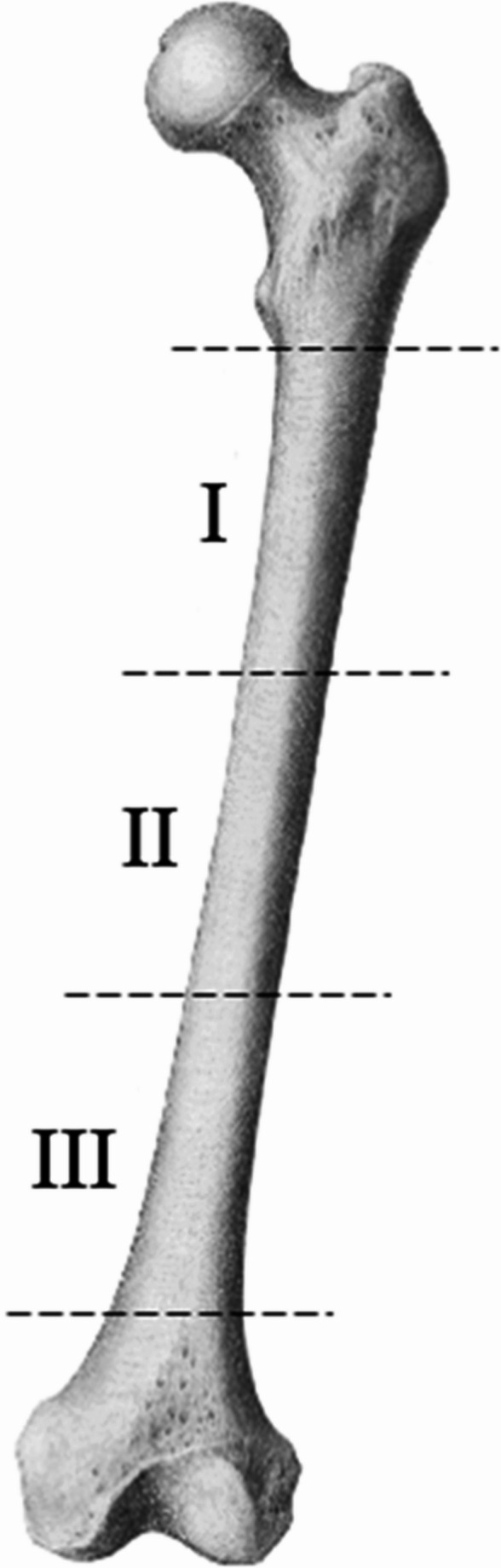
The shaft is divided in three segments (I: proximal shaft fracture, II: midshaft fracture, and III: distal shaft fracture). Only fractures of the middle segment of the shaft (II) were included in this analysis

The aim of this study was to compare the clinical outcome, the degree of radiographic malalignment, and the long-term patient-reported outcome in a series of consecutive patients with a fracture in the middle third of the femoral diaphysis (Figure 1), treated with either an antegrade or retrograde nail.

## Patients and methods

### Population

Adult patients (18 years or older) who sustained a fresh, traumatic fracture of the middle third part of the femoral diaphysis, treated with an intramedullary nail between January 1, 2001 and May 1, 2016 were enrolled in this retrospective cohort study. At least one postoperative radiograph showing the entire femur had to be available. Patients with a pathological fracture, a fracture in the proximal or distal third of the femoral shaft (type I and III, shown in Figure 1), patients who only underwent revision surgery in our facility, or patients with a periprosthetic fracture were excluded. Patients were asked to visit the outpatient department once for a long-term follow-up assessment. The local Medical Research Ethics Committee exempted this study (ref. no. MEC-2016-348).

### Treatment

All antegrade nailing has been performed in supine or lateral decubitus position. Fractures were reduced using a traction table or with manual traction to achieve adequate reduction. If necessary open reduction was performed. In case of a bent nail (Gamma-nail (Stryker, USA) or Holland Nail (Biomet, USA)) the aim was to use the greater trochanter as an entry point, for straight nails the aim was to use the piriformis fossa as an entry (T2 femur (Stryker, USA), ACE femoral nail (DePuy; Johnson& Johnson, USA), CFN (Synthes, Switzerland), or UFN (Synthes, Switzerland)). The retrograde nail is placed in supine position with the knee flexed in 30 degrees.

### Outcome and data collection

Data regarding patient characteristics, fracture characteristics, treatment details, and clinical outcome were collected from the patients’ medical files. Radiographs were analyzed using Digital Imaging and Communications in Medicine (DICOM) compliant viewer (RadiAnt DICOM version 3.2.2). The primary outcome was malunion which was assessed on the last available postoperative radiograph. Malunion is defined >5° angulation to the natural axis.

All patients were asked to visit the outpatient department once. Physical examination was performed by the first author who has not been involved in the treatment of any patient. It consisted of measurement of the rotational alignment and length of both femora and range of motion of the hip and knee. Rotational alignment was measured using the method described by Jaarsma *et al.* who showed thatclinical measurement of the rotation of the hip is a practical technique to assess rotational malalignment in patients after a femoral shaft fracture [17, 18].Leg length was measured using the direct tape measure method. In upright position, the distance between the posterior superior iliac spine and the floor, on bare feet, was measured. Duplicate measurements were averaged. An average leg length discrepancy of more than 2 cm was considered an axial malunion [19].

Patients were also asked to complete four patient-reported functional outcome questionnaires. The Western Ontario and McMaster Universities Arthritis Index (WOMAC), a disease-specific 24-item questionnaire, measuring three domains: pain, stiffness, and function. The WOMAC has been designed for patients with osteoarthritis of the hip and knee. A validated Dutch version was used [20]. The score ranges from 0 (worst outcome) to 96 (best outcome). The Lysholm Knee score contains eight items that are scored differently, using individual scoring scales [21]. The revised version of the Lysholm Knee Score was used. The score ranges from 0 (worst outcome) to 100 (best outcome). The Harris Hip Score (HHS) is a disease-specific test used to provide an evaluation system for various hip disabilities and treatments. It consists of a patient-reported part (pain (1 item; 44 points) and function (7 items; 47 points)), and a physician-reported part (range of motion (1 item; 5 points) and deformity (1 item; 4 points)) [22, 23]. The score ranges from 0 (worst outcome) to 100 (best outcome). The last questionnaire was the Dutch version of the Short Musculoskeletal Function Assessment (SMFA) [24]. It consists of a function index (39 items) and a bother index (14 items). The total score ranges from 0 to 100. A higher score on the SMFA indicates a worse outcome.

### Statistical analysis

Data were analyzed using the Statistical Package for the Social Sciences version 24.0 (Chicago, Ill., USA). Missing values were not replaced. Normality of continuous data was tested with the Shapiro-Wilk test, and homogeneity of variances was tested using the Levene’s test. Continuous data, which were all non-parametric, are presented as medians with first and third quartile. Categorical variables are provided as numbers and percentages. Statistical significance of differences were calculated with the Pearson Chi-squared test (for categorical variables) or Mann-Whitney U-test (for continuous variables). A p-value <0.05 was taken as a threshold of statistical significance.

## Results

### Study population

**Fig. 2:**
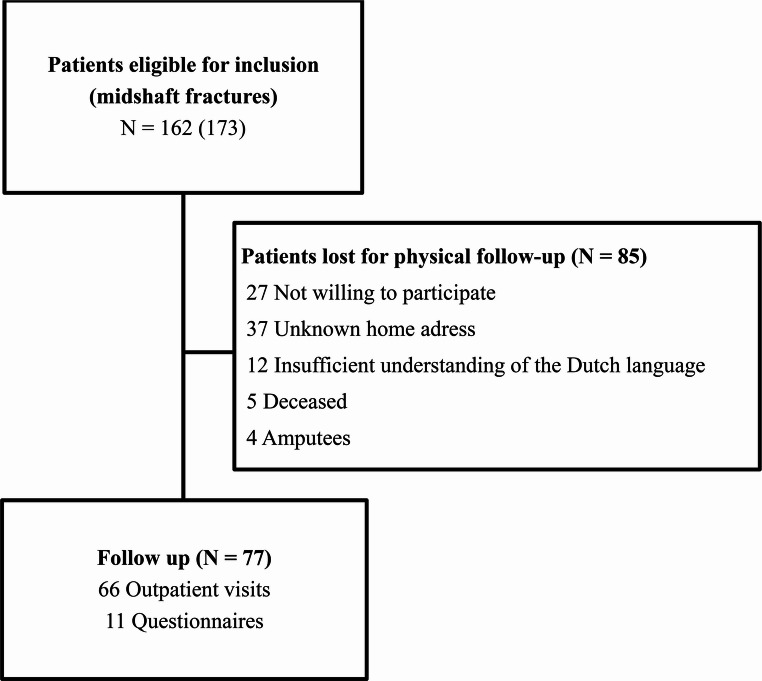
Flow chart patient selection

A total of 162 patients with 173 middle third shaft fractures were included for the primary outcome (Figure 2). Of the 173 fractures, 145 (84%) fractures were treated with an antegrade nail (AN group) and 28 (16%) were treated with a retrograde nail (RN group). The median time between trauma and revisiting the outpatient clinic was 9.0 (P_25_-P_75_4.4-12.1) years, with the longest being 15.7 years after trauma.

Of all patients 124 (77%) were male (Table 1). The median age was 28 years (P_25_-P_75_22-41 years). The median injury severity score was 13 (P_25_-P_75_9-23). Sixty (37%) patients were polytraumatized (ISS >15). Of the 162 patients, 112 (69%) had at least one additional injury. There was no statistically significant difference between the AN group and the RN group regarding demographic data, trauma characteristics, and fracture characteristics.

**Table 1:** Demographic data, trauma mechanism, and fracture characteristics at the time of injury in patients treated with antegrade and retrograde intramedullary nailing

		Total (*N* = 173)		Antegrade (*N* = 145)		Retrograde (*N* = 28)	*P*-value
	*N*		*N*		*N*		
***Patient characteristics***							
Age (years)	162	28 (22–41)	136	29 (22–28)	26	25 (22–39)	0.658
Male gender	162	124 (77%)	136	104 (77%)	26	20 (77%)	0.592
BMI (kg/m^2^)	137	24.8 (22.5–28.4)	116	24.8 (22.6–28.9)	21	23.1 (21.1–26.7)	0.142
ASA 1	156	114 (73%)		95 (73%)		19 (76%)	0.855
***Injury characteristics***							
Trauma mechanism							
HET, traffic	162	122 (75%)	136	104 (77%)	26	18 (69%)	0.116
HET, other		28 (17%)		24 (18%)		4 (15%)	
Injury Severity Score (ISS)	149	13 (9–23)	125	13 (9–23)	24	13 (10–25)	0.423
Polytrauma (ISS > 15)	149	60 (37%)	125	51 (41%)	24	9 (38%)	0.474
***Fracture characteristics***							
AO/OTA classification							
32-A	173	82 (53%)	145	76 (52%)	28	16 (57%)	0.847
32-B		63 (36%)		54 (37%)		9 (32%)	
32-C		18 (10%)		15 (10%)		3 (11%)	
Open fracture	173	43 (25%)	145	35 (24%)	28	8 (29%)	0.389

*BMI* Body Mass Index; *ASA* America Society Anesthesiologists score; *HET* High Energy Trauma; *LET* Low Energy Trauma; *N * number of patients. In the column in front of the data “N” indicates the number of patients of who data was available. Continuous data are shown as median (P_25_-P_75_), categorical data are shown as N (%)

Open reduction was performed in nine fractures (6%) of the AN group and twice in the RN group (7%); p=0.559. When antegrade nailing was performed the greater trochanter was used as an entry point in 87 (69%) of the fractures, whereas a corresponding bent nail was only used 55 fractures (63%). Remarkably, in the group with the greater trochanter as an entry point, patients treated with a straight nail showed no difference in the amount of varus or valgus. The median varus in patients treated with a straight nail was 3.0 degrees (P_25_-P_75_ 1.1-3.9) and with a bent nail 3.4 degrees (P_25_-P_75_ 1.3-4.1); p=0.693. Median valgus was respectively 2.5 degrees (P_25_-P_75_ 1.0-4.4) versus 3.4 degrees (P_25_-P_75_ 1.4-4.0); p=0.285.

### Complications and revision surgery

In the AN group 86 (59%) patients developed at least one complication *vs.* 13 (46%) in the RN group (p=0.146). Patients in the RN group developed more often a pneumonia (7 (25.0%) patients in the RN group *vs. *17 (11.7%) patients in the AN group; p=0.014). No statistically significant difference was observed in the rates of delayed union or nonunion between the groups (Table 2), although nonunion occurred in 8% of antegrade cases and 0% of retrograde cases.

**Table 2:** Most common complications postoperative complications and complications that occurred at initial follow-up in the outpatient clinic

Postoperative complications		Total (*N* = 173)		Antegrade (*N* = 145)		Retrograde (*N* = 28)	*P*-value
	*N*		*N*		*N*		
Number of patients with at least one complication	162	99 (61.1%)	136	86 (63.2%)	26	13 (50.0%)	0.146
Superficial infection	99	4 (2.3%)	86	4 (2.8%)	28	0 (0.0%)	0.564
Deep infection		5 (1.9%)		5 (3.4%)		0 (0.0%)	0.487
Pneumonia		24 (13.8%)		17 (11.7%)		7 (25.0%)	0.014
Other pulmonary complications (ARDS, pulmonary embolism)		8 (4.6%)		7 (4.8%)		1 (3.6%)	0.718
Delayed union		7 (4.0%)		6 (4.1%)		1 (3.6%)	0.639
Non-union		12 (6.9%)		12 (8.3%)		0 (0.0%)	0.165

N, number of patients. Continuous data are shown as median (P_25_-P_75_), categorical data are shown as N (%). ARDS, acute respiratory distress syndrome

No significant difference was seen between the two groups in rates of revision surgery on initial admission after trauma (Table 3). In the AN group 8 (15%) patients needed revision surgery vs. none of the patients of the RN group; p=0.353). Five of the patients required derotation due to (rotational) malalignment, the nail was removed in two patients and femoral plates were used for stabilization, and one patient required additional bone transplantation.

**Table 3:** Revision surgery during initial admission and during follow-up

	Total (*N* = 99)	Antegrade (*N* = 86)	Retrograde (*N* = 13)	*P*-value
Revision surgery				
Revision surgery at initial admission	8 (13.1%)	8 (14.8%)	0 (0.0%)	0.353
Changing IMN	5 (8.2%)	5 (9.3%)	0 (0.0%)	0.532
Bone transplantation	1 (1.6%)	1 (1.9%)	0 (0.0%)	0.885
Removal of IMN	2 (3.3%)	2 (3.7%)	0 (0.0%)	0.782
Dynamization of nail	10 (16.4)	9 (16.7%)	1 (14.3%)	0.678
Derotation osteotomy	3 (4.9%)	3 (5.6%)	0 (0.0%)	0.689
Removal of screws	10 (16.4%)	7 (13%)	3 (42.9%)	0.080
Removal of hardware after consolidation	24 (39.3%)	21 (38.9%)	3 (42.9%)	0.573

N, number of patients. Continuous data are shown as median (P_25_-P_75_), categorical data are shown as N (%). IMN, intramedullary nail. Percentages are reported as proportions relative to the total number of patients who experienced a complication (*N* = 99)

At follow-up no difference was noted between the groups in patients who needed dynamization of the femoral nail, patients who required a derotation osteotomy for excessive malalignment, and in the number of patients who required removal of the osteosynthesis. A trend toward significance was noted in the number of patients who experienced pain on the distal screws of the femoral nail (AN group 7 (13%) vs. RN group 3 (43%); p=0.080).

### Radiological outcome

The number of varus and valgus deformities in the AN and RN groups were similar. Recurvatum deformation was seen in 100 (70%) of the fractures in the AN group and 18 (64%) in the RN group. Table 4 shows the absence of any statistically significant difference between the AN and RN group.

**Table 4:** Radiological evaluation, angular malalignment based on the last available radiograph

	Overall(*N* = 173)		Antegrade (*N* = 145)			Retrograde(*N* = 28)			*P*-value
	*N*		*N*			*N*			
Degrees deformation									
Varus	89	2.1 (1.1–3.7)	75	2.1 (1.1–3.7)		14	2.3 (0.8–3.7)		0.706
Valgus	84	2.3 (1.0–4.0)	70	2.3 (1.0–4.0)		14	2.2 (1.4–4.6)		0.778
Recurvatum	118	3.0 (1.6–4.5)	100	3.0 (1.5–4.9)		18	3.1 (1.8–3.8)		0.866
Procurvatum	53	2.7 (1.5–4.0.5.0)	45	2.8 (1.4–4.0.4.0)		10	2.0 (1.6–8.9)		0.847

N, number of patients. Continuous data are shown as median (P_25_-P_75_), categorical data are shown as N (%)

### Range of motion of the hip and knee

Table5 shows the range of motion of the hip and knee of the injured side. Only the range of motion arc is shown. The median amount of external rotation possible, with the hip in extension, was higher in the antegrade nailing group than in the retrograde nailing group (45° (P_25_-P_75_30-45°)*versus*30°(P_25_-P_75_19-41°); *p*=0.023) (Table 5). Patients treated with a retrograde nail had a statistically significant lower flexion of the knee than patients treated with an antegrade nail (123° (P_25_-P_75_78-130°)*versus*130° (P_25_-P_75_120-140°); *p*=0.009). The flexion-extension range of the knee after RN was slightly, but statistically significantly lower than after AN (125° (P_25_-P_75_78-131°)*versus*130° (P_25_-P_75_130-140°); *p*=0.019).

**Table 5:** Range of motion of the hip and knee measured during physical examination at the outpatient clinic

		Overall (*N* = 66)		Antegrade (*N* = 56)		Retrograde (*N* = 10)	*P*-value
Range of motion	*N*		*N*		*N*		
***Hip***							
Flexion-Extension arc		160 (140–170)		160 (145–170)		150 (110–163)	0.135
Abduction-Adduction arc		80 (75–90)		80 (75–90)		80 (75–85)	0.371
90° ***flexed hip***							
Internal-external rotation arc		80 (70–90)		83 (70–90)		75 (27–90)	0.194
***Extended hip***							
Internal-external rotation arc		75 (64–90)		78 (66–90)		65 (19–79)	0.055
***Knee***							
Flexion-Extension arc		130 (120–135)		130 (130–140)		125 (78–131)	0.019

N, number of patients. Continuous data are shown as median (P_25_-P_75_), categorical data are shown as N (%)

N.D., P-value could not be determined. The outcome is shown in number of degrees

### Rotational malalignment

Rotational malalignment measured with a 90° flexed hip and knee was found in 23 (44%) patients (Table 6). Thirteen patients had an internal rotation deformity (median 15°; P_25_-P_75_ 10-23). Nine had an external rotational deformity (median 15°; P_25_-P_75_ 10-15°). This data shows no statistically significant difference in the number of rotational malalignment.

**Table 6:** Rotational malalignment measured during physical examination at the outpatient clinic

		Overall (*N* = 52)		Antegrade (*N* = 46)		Retrograde (*N* = 6)	*P*-value
Rotational malalignment	*N*		*N*		*N*		
Malalignment with hip in flexion		23 (44%)		21 (46%)		2 (33%)	0.453
Internal rotation (º)	13	15 (10–23)	12	15 (10–26)	1	15 (15–15)	0.923
External rotation (º)	10	15 (10–15)	9	15 (10–15)	1	10 (10–10)	0.400
Malalignment with hip in extension		25 (48%)		23 (50%)		2 (33%)	0.372
Internal rotation (º)	18	15 (10–15)	16	15 (10–15)	2	15 (15–15)	0.641
External rotation (º)	7	15 (10–15)	7	15 (10–15)	0	0 (0–0)	N.D.

N, number of patients. Continuous data are shown as median (P_25_-P_75_), categorical data are shown as N (%)

N.D., P-value could not be determined

### Leg length discrepancy

Three patients sustained leg length discrepancy >1 cm in upright position, two with 1.3 cm and one with 3.5 cm, all the result of shortening of the injured leg. A statistically significant difference in LLD between treatment groups was absent.

### Patient-reported functional outcome scores

A total of 70 patients completed questionnaires and physical examination. For the LKS 77 fractures were addressed for evaluation, total score was based on 70 patients. Only the total scores of the questionnaires are shown. The WOMAC revealed no difference between antegrade and retrograde nailing (median 93 points (P_25_-P_75_ 75-100) *versus* 85 points (P_25_-P_75_59-97) respectively; *p*=0.289; Table 7). The Harris Hip Score showed an overall good score, with a total score of 93 points (P_25_-P_75_85-97) for AN group *versus* 91 points (P_25_-P_75_71-97) for the RN group (*p*=0.470). The AN group showed a better knee function on the LKS than the RN group (83 (P_25_-P_75_68-95) *versus*61 (P_25_-P_75_48-84); *p*=0.041). The AN group also reported less disability on the SMFA dysfunction index than the RN group (13 points (P25–P75: 5–23) versus 29 points (P25–P75: 9–43); p = 0.026).

**Table 7:** Patient-reported outcome measures in patients who completed the questionnaires

		Overall (*N* = 70)		Antegrade (*N* = 60)		Retrograde (*N* = 10)	*P*-value
	*N*		*N*		*N*		
***WOMAC***							
Total	70	92 (75–99)	60	93 (75–100)	10	85 (59–97)	0.286
***Harris Hip Score***							
Total	65	93 (84–97)	56	93 (85–97)	9	91 (71–97)	0.470
***Lysholm Knee Score***							
Total	77	81 (63–95)	66	83 (68–95)	11	61 (48–84)	0.041
***Short Musculoskeletal Function Assessment***							
Dysfunction index	70	14 (5–26)	60	13 (5–23)	10	29 (9–43)	0.026
Bother index		14 (4–29)		14 (4–27)		21 (6–47)	0.305
Total		15 (5–25)		14 (5–23)		27 (8–43)	0.054

N, number of patients; WOMAC, Western Ontario and McMaster Universities Arthritis index. Continuous data are shown as median (P_25_-P_75_), categorical data are shown as N (%). The Harris Hip Score contains a patient-reported part and a physician-reported part, patients who only fulfilled the questionnaires were excluded in the definitive calculation of the questionnaires

## Discussion

This study compared the outcome of antegrade and retrograde nailing of middle-third shaft fractures of the femur over a 15 years period in a level 1 trauma center. Since healing of the fracture was not a problem, emphasis was put on malunion and functional outcome. Although small angular deviations were frequent, no radiological difference was found in angular malalignment between retrograde and antegrade nailing. Rotational malalignment was found in approximately half of all patients, but it did not exceed 15°, irrespective of the nailing technique used. Comparison of patient-reported outcome regarding function of the hip with the WOMAC and HHS showed no difference between antegrade and retrograde nailing. Patients treated with an antegrade nail showed a higher range of motion of the knee compared to retrograde nailing. Also the Lysholm Knee Score performed better in the AN group as compared to the RN group. The AN group scored better on the function index of Short Musculoskeletal Function Assessment than the RN group. Although the absolute numbers in the subgroups are small, the results all show the same trend: retrograde nailing is not without a price to knee function, whereas antegrade nailing seems to spare the function of the hip.

Little is known about the effect of angular deformity after a femoral shaft fracture. Angular malalignment >5° is considered clinically relevant as it may trigger complaints like knee pain and discomfort during daily activities [25, 26] and justify surgical correction. Even though the greater trochanter was used as the entry point in 62% and only in 34% of the fractures a corresponding bent nail was used, the overall degree of substantial angular malalignment in the population suffering from a middle third shaft fracture was low. This might be explained by the fact that the location of the fracture and diameter of the intramedullary canal, combined with a relatively small diameter of the straight nails used, allowed the surgeon to direct the tip of the nail into the medial distal femoral condyle instead of the intercondylar area. This might be different in patients with an AO/OTA type 32 fracture above the isthmus. In such injuries the nail will push the proximal fragment into varus with concomitant loss of leg length. Most fractures healed in recurvatum (118 (68%) but without serious malunion. No malunions >15° were found in any plane. This is consistent with current literature showing a rate of angular deformation >15° ranging from 0-11% [9, 15, 25, 27]. The rate of rotational malalignment was surprisingly high. However, the number of serious rotational malalignment (>15°) in this study was low. These numbers are also in concordance with literature [17, 28, 29]. On the other hand, if the goal of osteosynthesis is restoration of anatomy and uneventful fracture healing, any deviation of that aim implicates that room for improvement in both surgical technique and implant design exists. The given definitions of malalignment are just thresholds for secondary surgical correction.

Although various results in patient-reported outcomes after a femoral shaft fracture have been described, only few studies have examined long-term functional outcomes. Previous literature showed no difference on the Lysholm Knee Score, and muscle function between antegrade and retrograde nailing after one year of follow up [16]. Equal functional scores on the Short Musculoskeletal Function Assessment were found in a series of patients with distal femoral shaft fractures when antegrade and retrograde nailing were compared [30]. With a follow up of nearly 8 years, no differences were found in range of motion and no differences on the same questionnaires used in this study when antegrade nailing was compared with retrograde nailing for femoral shaft fractures [31].

The current study is in contradiction with other studies that show similar clinical results after either antegrade nailing or retrograde nailing of any femoral shaft fracture [16, 30–32]. This might at least in part be explained by the fact that we only included true midshaft femoral fractures to avoid independent confounding variables that could relate to knee or hip pain or mechanical advantages dictating the need for one specific technique. This study showed that antegrade intramedullary nailing of femoral midshaft fractures resulted in a higher range of motion of the knee, less discomfort on the LKS, and higher function index of the SMFA than patients treated with retrograde intramedullary nailing. Even though no big axial malunion was seen in the radiographic analysis, retrograde nailing may cause discomfort in the knee due to damage to the articular tissue.

The current study has several limitations. First, as a result of the retrospective design the exact reason for retrograde or antegrade nailing was not always clear. Moreover, since retrograde nailing was hardly performed in the early years of the long inclusion period, an unequal distribution of both techniques was observed. A prospective and randomized design would reduce inclusion bias.

A substantial number of participants completed the follow-up, which included four validated questionnaires and a comprehensive physical examination. The strengths of this study include the large sample size for assessing malalignment and the extended duration of follow-up, with a median of 9.0 years post-trauma and a maximum of 15.7 years post-trauma. But the low response rate to the questionnaire (45%) suggests the potential for response bias. But the key limitations of this study is the discrepancy in sample sizes between the subgroups, particularly the smaller sample size in the RN group (N=10) compared to other subgroups. This imbalance may have impacted the statistical power of our analyses and the generalizability of our findings. Given this limitation, the results related to the retrograde nails subgroup should be interpreted with caution. While our findings provide valuable insights, the small sample size may limit the strength of any conclusions regarding clinical and functional outcomes. Future studies with larger and more balanced sample sizes are necessary to confirm these results and to ensure more robust and generalizable conclusions. Additionally, we acknowledge that the discrepancy in sample sizes could have warranted a correction to the statistical tests employed, such as adjustments for unequal variances or the application of more conservative significance thresholds. However, even with these considerations, the limited sample size of the retrograde nails group poses challenges in making definitive statements about its clinical implications.

Several methodological limitations should be acknowledged. Although the threshold was chosen based on clinical relevance and earlier research, the categorization of alignment data as either smaller than or greater than 5 degrees from 'normal' is a simplification that may overlook nuances in the data. And last, the variability in the time from injury to the follow-up X-ray is another important consideration. In this retrospective study, the time frame between injury and radiographic assessment was not standardized, which could introduce some variability in the results. This variability may have influenced the findings and should be accounted for in the interpretation of the results.

## Conclusion

This study showed good technical outcomes after surgical treatment of a midshaft femoral fracture with low percentages of angular and rotational malalignment in both antegrade and retrograde nailing groups. Antegrade nailing and retrograde nailing yielded similar overall results. Although the complication rates between groups did not differ significantly, the retrograde group showed fewer rotational issues, and the antegrade group demonstrated slightly better patient-reported outcome measures. Based on the current results, we suggest considering antegrade nailing over a transarticular retrograde approach for the treatment of middle third femoral fractures, if technically feasible.

## Data Availability

This research received no specific grant from any funding agency in the public, commercial or not-for-profit sectors. There are no statements to declare relating contributorship, data sharing, or ethics approval.
